# On Modern Progress in Ophthalmic Medicine and Surgery

**Published:** 1894-07-07

**Authors:** Robert Brudenell Carter

**Affiliations:** Consulting Ophthalmic Surgeon to St. George's Hospital


### On Modern Progress in Ophthalmic Medicine and Surgery.

By Robert Brudenell Carter, F.R.C.S., Consulting Ophthalmic Surgeon to St. George's Hospital.

SENILE CATARACT (*Continued*).

In a series of papers intended only to afford an outline of progress, and not, even collectively, to constitute a complete treatise, it is unnecessary to deal at any length with the complications and difficulties which occasionally present themselves in an operation for senile cataract, or in the course of the subsequent recovery, The most important complication likely to arise, during the operation itself, is loss of vitreous humour, either before or after the extrusion of the lens; but this, which was once a not uncommon consequence of reflex muscular spasm, can scarcely occur, now that the stimulus exciting to such spasm has been removed by the use of cocaine, except from misdirected or excessive pressure on the part of the surgeon. The most important complication likely to arise during the progress of recovery is delayed healing of the incision, either with or without, but most commonly with, a certain degree of iritis. It has recently been alleged that the free application of cocaine, that is to say, its use in quantity sufficient to produce insensibility of the iris as well as of the cornea, so modifies the corneal tissue as to render it indisposed to heal; but, when it is considered that the subjects of the operation are generally persons far advanced in life, and that failure to heal, even under complete antiseptic precautions, was not unknown before cocaine was introduced, it is obvious that more evidence than is at present forthcoming would be required in order to establish such a position, or to justify the surgeon in allowing a patient to suffer unnecessary pain, with its attendant risks of muscular spasm. Delayed healing is most prone to occur in marasmic persons, and especially when there is a thin and feeble cornea, which, upon the extrusion of the lens, completely loses its convexity, and either falls into wrinkles or into a saucer-like depression. Such a condition is manifestly unfavourable to the first condition of prompt healing, that is to say, to the maintenance of the lips of the incision in perfect contact. However produced, delayed healing is always liable to be followed by chronic inflammation, which, unless promptly arrested, is always injurious, and may even lead to complete wasting of the eyeball and loss of sight. It is best dealt with by keeping the lids closed, by the daily instillation of a drop of a solution of atropine and cocaine, and, above all, by the liberal administration of alcohol.

In some cases, either from threatened loss of vitreous, or even merely from not seeing portions of the cortex of the lens which are still transparent, the surgeon may leave a certain amount of this cortex within the eye. Such cortex is prone to set up iritis, and may also lead to increase in the quantity of the residual material, an increase due either to the effusion of lymph or to cell proliferation. In the period of the flap operation, such an occurrence was almost invariably followed by suppuration of the eyeball; but, in the present day, the inflammation is usually controllable, and the ultimate results may be excellent. The pupil should be kept fully dilated, after the fourth day, by the application, at least twice a day, of a drop of a solution contraining two grains of atropine and four of cocaine to the ounce; and the eye may be fomented or bathed three or four times daily, with a cup sponge wrung out of hot water. The most important part of the treatment, however, consists in the repeated application of a leech every day or every other day, according to the severity of the case, and until recovery is well advanced. The leech should be applied on the same horizontal line as the opening of the eyelids, and as far away from the outer angle of this opening as the growth of hair will allow. When the eye has otherwise recovered, and is again white and with good perception of light, the pupil will generally be obstructed by a residual film adhering to its margin, filling the gap left by the iridectomy, and, of course, interfering with vision. The best way of dealing with such a film is to tear a central opening in it, using two needles in the way first described by the late Sir William Bowman. The little operation should be done under cocaine, and the eye should be kept closed for some twenty-four hours afterwards. After the lapse of a week it will generally be ready to be fitted with its appropriate glasses. In doing this, care should be taken not only to supply the necessary spherical lenses, but also to examine for astigmatism, and to correct any defect of this kind which may be discovered.

There has lately been a considerable tendency, on the part of ignorant people in this country, to extol the work of German ophthalmologists, and to depreciate what has been done in England. The tendency has even been exalted into a "fashion," which has induced many English people to resort to Germany for treatment, seldom, it may be observed, at the hands of the really eminent German ophthalmologists, the professors and teachers at important seats of learning, but usually at those of persons practising at health resorts, and whose pretensions have been made known by systematic oblique advertisement, mainly conducted by syndicates of hotel keepers. The foregoing papers will enable all who read them to form a fair estimate of the relative merits of English and German ophthalmologists with regard to the one subject of cataract extraction. After a century without advancement, the first step forwards was taken in Germany, under the stimulus of the extreme badness of the results previously obtained there. Von Graefe suggested that improvement was possible, and pointed to the direction in which it should be sought; but the precise line of incision which he indicated has been abandoned. He also suggested, and Mooren carried out, the employment of iridectomy as a preliminary proceeding. The other German suggestions for improvements in the methods of operating have perished under the weight of their own futility. The departure from Von Graefe's incision, which is now generally adopted in all countries, is that which was arrived at by the late Sir William Bowman, and the late Mr. Critchett, as the result of their work at Moorfields. Antiseptic surgery, the application of which to the eye has produced the most brilliant results, is, it need hardly be said, entirely English. The practical application of cocaine is due to Austria. Hence, if we take a general survey of the question, we shall find it difficult to escape from the conclusion that both countries have contributed, in almost equal proportions, to the general result, and that neither can claim any pre-eminence over the other. The knowledge possessed in either is common to both, and the only differences which exist among ophthalmic surgeons are not between English and Germans, but between individuals who may be gifted with varying degrees of aptitude in the application of principles with which all are alike familiar.

